# Combined treatment of miltefosine and paromomycin delays the onset of experimental drug resistance in *Leishmania infantum*

**DOI:** 10.1371/journal.pntd.0005620

**Published:** 2017-05-15

**Authors:** Sarah Hendrickx, Magali Van den Kerkhof, Dorien Mabille, Paul Cos, Peter Delputte, Louis Maes, Guy Caljon

**Affiliations:** Laboratory for Microbiology, Parasitology and Hygiene (LMPH), University of Antwerp, Antwerp, Belgium; Hebrew University-Hadassah Medical School, ISRAEL

## Abstract

**Background:**

Since miltefosine monotherapy against visceral leishmaniasis (VL) caused by *Leishmania donovani* has been discontinued in the Indian subcontinent due to an increase in the number of treatment failures, single dose liposomal amphotericin B is now advocated as a treatment option of choice. Paromomycin-miltefosine combination therapy can be used as substitute first-line treatment in regions without cold-chain potential. Previous laboratory studies in the closely related species *Leishmania infantum* have demonstrated that paromomycin monotherapy fairly rapidly selects for resistance producing a phenotype with increased fitness. Given the possible clinical implications of these findings for the current field situation, the present study aimed to identify the potential hazards of paromomycin-miltefosine combination therapy.

**Principal findings:**

Drug interaction studies using the fixed-ratio isobologram method revealed an indifferent interaction between paromomycin and miltefosine. In hamsters infected with *L*. *infantum*, the combination resulted in cumulative efficacy in reducing parasite burdens in the liver, spleen and bone marrow. Selected resistant lines against the single drugs did not display cross-resistance. When the intracellular amastigote stage was repeatedly exposed to the paromomycin-miltefosine combination, either *in vitro* or *in vivo*, no significant susceptibility decrease towards either drug was noted.

**Conclusions:**

These results suggest that implementation of paromomycin-miltefosine combination therapy indeed could represent a safe and affordable treatment option for *L*. *donovani* VL as miltefosine appears to overrule the anticipated rapid development of PMM resistance.

## Author summary

Liposomal amphotericin B is presently being used as first-line treatment option against visceral leishmaniasis in the Indian subcontinent. However, the need for temperature-controlled transport and storage limits its widespread use in rural areas. Previous studies already suggested that paromomycin-miltefosine combination therapy could be a valuable alternative, side passing some of the disadvantages associated with monotherapy, such as development of drug resistance. As the first reports of miltefosine resistant clinical isolates have already surfaced and paromomycin resistance could be easily induced under laboratory conditions, it remains essential to assess the risk of developing resistance against both drugs upon combination therapy. This study evaluated the efficacy of combined therapy against a *Leishmania* species closely related to the agent found in the Indian subcontinent, using both *in vitro* and *in vivo* models with the aim to select multidrug-resistant species by simultaneous exposure to paromomycin and miltefosine. The combination of both drugs in the hamster model resulted in a cumulative efficacy but did not lead to a significant susceptibility decrease, indicating that paromomycin-miltefosine combination therapy may represent a safe and affordable treatment option for visceral leishmaniasis.

## Introduction

Depending on the geographical location, visceral leishmaniasis (VL) can either be caused by *Leishmania donovani* (East Africa and Indian subcontinent) or *L*. *infantum* (Mediterranean basin, central Asia and Latin America) [1]. Although both species are closely related and belong to the same complex (*L*. *donovani* complex), transmission of *L*. *donovani* is mostly anthroponotic while the domestic dog serves as the main reservoir of *L*. *infantum*. Various treatment recommendations for VL have been proposed in the past and up to the early 2000’s most cases were treated with antimonials (Sb) [2]. However, the spread of Sb-resistant parasites in the Indian subcontinent has enforced a shift in therapeutic modalities [3]. Given its limited toxic effects, oral administration and reasonable price, the introduction of miltefosine (MIL) in 2002 as a novel antileishmanial agent looked very promising. As a result, the drug was even presented as first-line therapy in 2005 in India, Bangladesh and Nepal in the frame of the Kala-azar elimination program aimed at reducing the disease burden to less than 1/1000 by 2015 [4]. Unfortunately, recent studies in the Indian subcontinent demonstrated an increasing number of MIL-treatment failures hence limiting its further use in monotherapy [5] and leading to the recommendation of a single dose of liposomal amphotericin B (L-AmB) as treatment option of choice [6, 7]. However, the requirement for temperature-controlled cold-chain facilities to transport and preserve L-AmB restricts its widespread use. In regions without cold-chain assurance, the short-term combination of MIL and paromomycin (PMM) has been suggested as alternative [8]. The development of MIL-resistance in monotherapy can be anticipated as a major limitation given its long elimination half-life and long treatment course [9]. Although no decreased MIL-susceptibility could yet be demonstrated *in vitro* in isolates of *L*. *donovani* derived from treatment failures in the Indian subcontinent, the first actual MIL-resistant clinical *Leishmania* isolates did already surface [10–12]. Rapid development of laboratory-induced PMM-resistance has been demonstrated [11, 13–15] which also proved to be associated with a potential fitness gain [16, 17] paving a way to rapid emergence and spread of PMM-resistance upon its routine use in the field. Although drug resistance would theoretically arise slower for drug combinations, it remains essential to assess the potential effect of introducing PMM-MIL combination therapy on a large scale, given the above mentioned limitations of PMM- or MIL-monotherapy. This laboratory study assessed the interaction between MIL and PMM *in vitro* and *in vivo*, the occurrence of cross-resistance and the development of resistance upon repeated drug exposure cycles to this drug combination.

## Materials and methods

### Ethics statement

The use of laboratory rodents was carried out in strict accordance to all mandatory guidelines (EU directives, including the Revised Directive 2010/63/EU on the Protection of Animals used for Scientific Purposes that came into force on 01/01/2013, and the declaration of Helsinki in its latest version) and was approved by the ethical committee of the University of Antwerp, Belgium [UA-ECD 2011–77 (17-02-2012)].

### Animals

Female Swiss mice (20–25 g) and female golden hamsters (80–100 g) were purchased from Janvier (France). Food for laboratory rodents (Carfil, Arendonk, Belgium) and drinking water were available *ad libitum*. Hamsters were kept in quarantine for at least 5 days before infection and were randomly allocated to experimental units of 5 animals each, with the exception of the groups (*n* = 3) for the selection of resistance.

### *Leishmania* parasites

The *L*. *infantum* laboratory strain ITMAP263 (MHOM/MA/67/ITMAP263) was routinely cultivated in Syrian golden hamsters. To infect naive hamsters, *ex vivo* amastigotes were purified from the spleen of heavily infected donor hamsters, as previously described [14]. Infection inoculates containing 2 x 10^7^ amastigotes/100 μL phosphate buffered saline (PBS) were used to infect hamsters by intracardial injection under isoflurane inhalation anaesthesia. The general condition and body weight of infected animals were monitored daily to evaluate the course of infection. Upon each drug resistance selection cycle, promastigote back-transformation was performed to allow *in vitro* expansion of surviving parasites for *in vitro* susceptibility determination purposes.

Promastigotes of the *L*. *infantum* clinical isolate LEM3323 (MHOM/FR/96/LEM3323) were obtained from the ‘Centre National de Référence des Leishmania (CNRL)’ (Dr. L. Lachaud) and derived from a French HIV patient. Promastigotes were maintained in HOMEM promastigote medium supplemented with 10% inactivated fetal calf serum (Invitrogen, Ghent, Belgium). To facilitate promastigote back-transformation from infected tissues during *in vivo* selection of resistance, 20% spent promastigote medium was added and the concentration of inactivated fetal calf serum was augmented to 20% [14].

### Drug formulations and preparation

Both MIL (MW = 407.57) and PMM-sulphate (MW = 713.71) were purchased from Sigma (Diegem, Belgium). For the *in vitro* work, stock solutions of 20 mM were prepared in PBS (MIL) or distilled water (PMM). For the treatment of infected animals, MIL and PMM were formulated in distilled water respectively at 20 mg/mL and 150 mg/mL.

### Amastigote susceptibility determination *in vitro*

*In vitro* amastigote susceptibilities were determined as previously described [18]. To determine the drug susceptibility of intracellular amastigotes, primary peritoneal macrophages were harvested from starch-stimulated female Swiss mice. Forty-eight hours later, cells were infected with metacyclic promastigotes at an infection ration of 2:1 (LEM3323) or 20:1 (ITMAP236). After 24h, the medium was changed to remove potential extracellular promastigotes. The plates were incubated at 37°C in 5% CO_2_ atmosphere for another 5 days in presence of 2-fold drug dilutions before staining with Giemsa and microscopic reading.

### *In vitro* efficacy evaluation of MIL-PMM combinations

**Fig 1:**
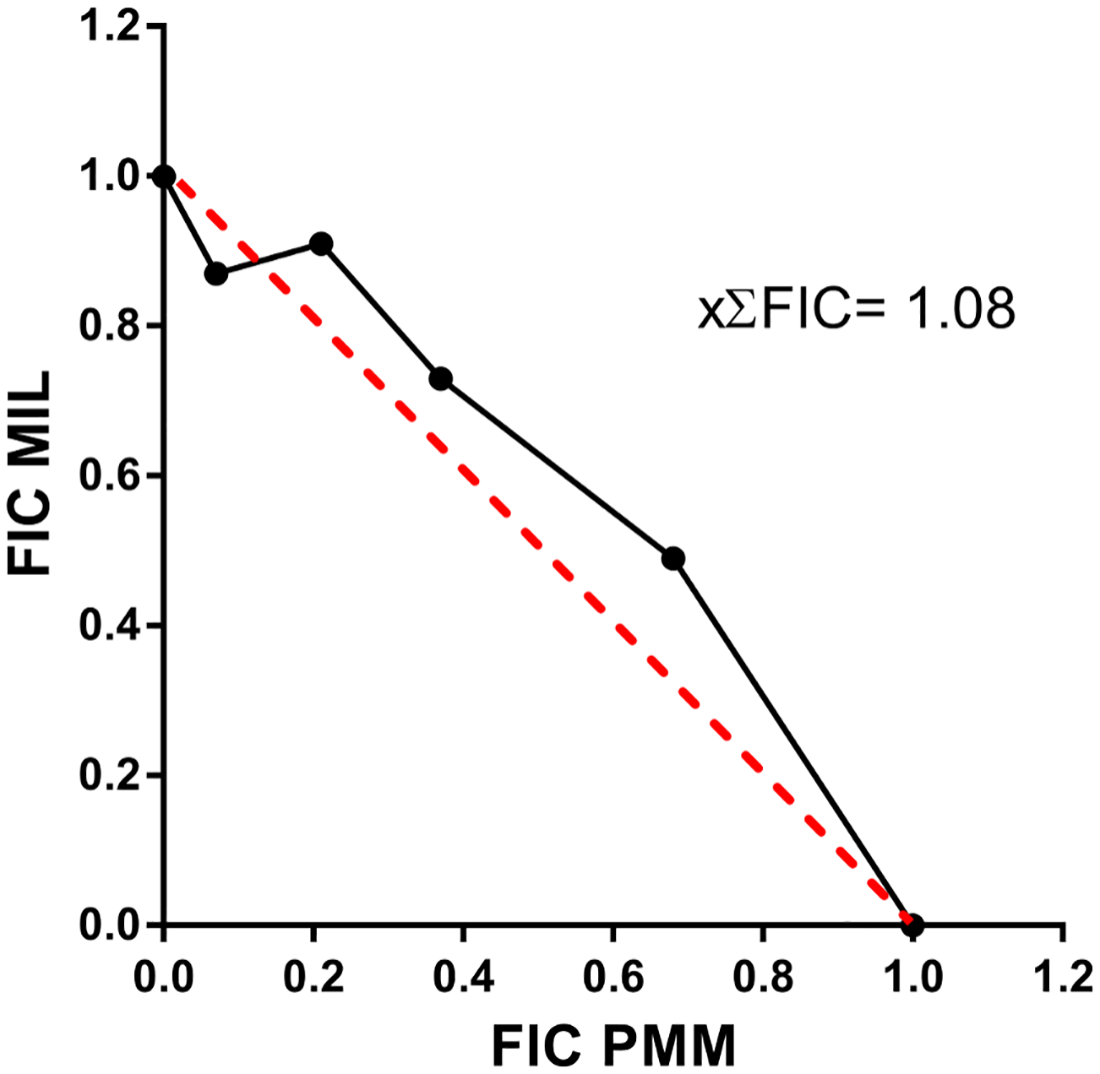
Isobologram of the interaction of PMM-MIL combination against intracellular amastigotes *in vitro* at the IC_50_ level. For each fixed-ratio, normalized fractional inhibitory concentrations (FICs) are presented for MIL on the y-axis and for PMM on the x-axis. The constructed isobologram is the result of 4 independent experiments run in duplicate.

The normalized fixed ratio isobologram method [19] was used to evaluate the efficacy of the combination of PMM and MIL against the *L*. *infantum* clinical isolate (LEM3323) *in vitro* at their IC_50_ level. Intracellular amastigotes were exposed to 2-fold dilution series of the drug combinations in fixed drug ratios (5:0, 4:1, 3:2, 2:3, 1:4, 0:5). Top concentrations of MIL and PMM were selected based on their known susceptibility profiles with the intention to center the IC_50_ of each respective drug in the middle of a seven-point two-fold drug dilution series. For MIL, a top concentration of 5 μM was used while for PMM 500 μM was selected. As in the standard susceptibility assay, drug exposure lasted for 5 days where after the plates were stained with Giemsa and the susceptibility (IC_50_) towards each drug was determined for each combination ratio. Fractional inhibitory concentrations (FICs) were determined by dividing the IC_50_ of the drug in combination by the IC_50_ of the drug alone and were used to construct the isobologram. The sum of FICs (∑FICs) (FIC MIL + FIC PMM) was determined for each fixed-ratio solution and the mean ∑FIC was used to classify the nature of the drug interaction (Fig 1). Interactions are classified synergistic when the mean ∑FIC < 0.5, indifferent when the mean ∑FIC ranges between 0.5 and 4 and antagonistic as the mean ∑FIC > 4.

### *In vivo* efficacy evaluation of MIL-PMM combination therapy

Before defining the appropriate treatment regimen for the selection of drug resistance *in vivo*, the efficacy of the envisaged treatment regimens either combined or alone was evaluated. Hamsters were infected with *ex vivo* ITMAP263 amastigotes as described above. At 21 days post-infection (dpi), animals were treated with either MIL, PMM or the combinations, as listed in Table 1. Due to toxicity, the combination 350 PMM/kg and 40 mg MIL/kg was not included. At 35 dpi, the animals were sacrificed to assess parasite burdens in liver, spleen and bone marrow. Upon fixation in methanol and Giemsa-staining, the liver and spleen imprints and bone marrow smears were evaluated microscopically by counting the number of amastigotes associated with 500 macrophage nuclei. To evaluate drug efficacy, the percentage reduction compared to the untreated control group was calculated.

**Table 1:** Efficacy of PMM-MIL combination therapy *in vivo*. Percentage reduction in amastigote burdens (*L*. *infantum* ITMAP263) in liver, spleen and bone marrow compared to the vehicle treated infected control (VIC) group.

Dosing group: treatment regimen	% reduction of amastigote burdens in target organs		
	Liver	Spleen	Bone-marrow
G1: VIC	-	-	-
G2: MIL - 40mg/kg PO—s.i.d. x 5 days	95.3	99.4	86.8
G3: MIL—20 mg/kg PO—s.i.d. x 5 days	80.0	94.5	76.9
G4: MIL– 10 mg/kg PO—s.i.d. x 5 days	18.9	54.8	48.1
G5: PMM—180 mg/kg IP—s.i.d. x 5 days	79.9	64.4	0.0
G6: PMM—350 mg/kg IP—s.i.d. x 5 days	85.1	74.4	84.5
G7: MIL—20 mg/kg PO—s.i.d. + PMM—350 mg/kg IP—s.i.d. x 5 days	99.6	99.6	98.1
G8: MIL—10 mg/kg PO—s.i.d. + PMM—180 mg/kg IP—s.i.d. x 5 days	97.3	96.0	88.0
G9: MIL—20 mg/kg PO—s.i.d. + PMM—350 mg/kg IP—s.i.d. x 2 days	59.3	56.1	32.5

### Selection of resistance *in vitro*

To check whether drug resistance would arise upon PMM-MIL combination, resistance was selected *in vitro* in the LEM3323 isolate as described earlier [13]. The highest combined drug concentrations were 500 μM PMM and 40 μM MIL. The drug susceptibility was evaluated *in vitro* after each selection cycle. The selection process was performed for five successive cycles or until resistance towards PMM (IC_50_ >150μM) or MIL (IC_50_ >15μM) was reached [20].

### Selection of resistance *in vivo*

Resistance against the MIL-PMM combination therapy was also selected *in vivo*, as described earlier [14]. Infected hamsters were repeatedly exposed to sub-curative and sub-toxic doses of the drug combination (*i*.*e*. oral administration of MIL at 20 mg/kg and intraperitoneal injection of 350 mg PMM/kg for 2 days). Infected animals were treated for 2 days starting from 21 dpi and closely monitored for treatment relapse. When treatment relapse was suspected, a liver biopsy was taken to quantify the infection burden and to enable susceptibility testing of the drug-exposed parasites upon promastigote back-transformation. Maximum two subsequent treatment cycles were performed in the same animal before *ex vivo* amastigotes were harvested and transferred to a next naïve animal. Subsequent selection rounds were terminated either when drug susceptibility values indicated a drug-resistant phenotype (IC_50_ MIL >15μM and IC_50_ PMM >150μM) or after a maximum of 5 successive treatment/relapse cycles.

## Results

### *In vitro* efficacy evaluation of MIL-PMM combinations

For the construction of the isobologram (Fig 1) and determination of drug interaction on the LEM3323 isolate by the applied fixed-ratio isobologram method, four independent assays were performed, each in duplicate. the mean ∑FIC was 1.1 ± 0.3 at the IC_50_ level with ∑FICs ranging between 0.94 and 1.17, indicating an indifferent interaction between MIL and PMM.

### *In vivo* efficacy evaluation of MIL-PMM combination therapy

Results of the *in vivo* efficacy study are represented in Table 1. Combination of PMM-MIL for 5 days showed a superior efficacy compared to monotherapy with PMM or MIL at either treatment regimen used. MIL reached higher efficacies in clearing the splenic parasite burden, whereas PMM seemed more efficacious against the hepatic parasite burden. Combination of both drugs resulted in splenic and hepatic reductions of >99% and >98% in the bone marrow at the highest dosage scheme. Shortening the duration of the combination therapy from five to two days resulted in a significant efficacy drop with reductions of <60% in the liver and spleen and only 32.5% in the bone marrow.

### Selection of resistance *in vitro*

Results of the *in vitro* resistance selection are presented in Table 2. Despite a shift in promastigote back-transformation following exposure to the PMM-MIL combination, allowing collection of surviving promastigotes exposed to increasing drug concentrations, no differences were observed between the susceptibility results of the wild-type strain and the strain that was repeatedly exposed PMM and MIL. Also no cross-resistance could be detected in the MIL and PMM resistant lines.

**Table 2:** Amastigote susceptibility (IC_50_) of the *in vitro* resistance selection procedure to PMM-MIL combination therapy. No difference was observed between IC_50_ of the wild-type strain (LEM3323 WT) and the strains that underwent three (LEM3323 PMM/MIL3) or five (LEM3323 PMM/MIL5) repeated exposures to the combined high concentrations of PMM and MIL. Exposure to 5 treatment cycles with either PMM (LEM3323 PMM) or MIL alone (LEM3323 MIL) revealed a clear decrease in drug susceptibility (indicated in bold) [21].

Strain	Drug susceptibility (μM)	
	Paromomycin (PMM)	Miltefosine (MIL)
LEM3323 WT	98.0 ± 14.3	1.0 ± 0.1
LEM3323 PMM/MIL3	109.5 ± 26.0	0.7 ± 0.2
LEM3323 PMM/MIL5	64.3 ± 13.6	0.8 ± 0.4
LEM3323 PMM	**212.6 ± 30.9**	0.5 ± 0.1
LEM3323 MIL	68.5 ± 8.3	**> 20.0**

### Selection of resistance *in vivo*

Five subsequent treatment/relapse cycles were conducted and despite recurrent relapses, no resistant phenotype could be observed for either MIL or PMM (Table 3). In contrast, exposure to PMM alone resulted in a significant decrease in susceptibility against PMM without altered MIL resistance profile.

**Table 3:** Amastigote susceptibility results of the *in vivo* resistance selection procedure to PMM-MIL combination therapy. No difference was observed between IC_50_ of the wild-type (ITMAP263 WT) and the strain that was exposed five times to high concentrations of PMM and MIL (ITMAP263 PMM/MIL). Exposure to PMM alone (ITMAP263 PMM) revealed a significant susceptibility decrease (indicated in bold)[14]. (ND: not done).

Strain	Drug susceptibility (μM)	
	Paromomycin (PMM)	Miltefosine (MIL)
ITMAP263 WT	135.8 ± 41.0	2.3 ± 0.3
ITMAP263 PMM/MIL	123.1 ± 23.6	3.2 ± 0.1
ITMAP263 MIL	ND	3.0 ± 0.3
ITMAP263 PMM	**430.9 ± 24.5**	3.5 ± 0.4

## Discussion

In response to the recent development and spread of clinical Sb-resistance in *L*. *donovani*, other drugs such as MIL, PMM and AmB have been proposed for VL treatment. Nowadays, a single dose of liposomal AmB has been recommended as first-line treatment in the Indian subcontinent [6], however, despite the initiative from Gilead of donating a large batch of liposomal AmB, the need for temperature-controlled settings still severely limits its widespread use. Moreover and comparable to MIL, the first cases of AmB unresponsiveness have recently been reported [22, 23]. In those endemic areas with large-scale Sb-resistance and an increasing number of MIL and AmB treatment failures, a switch from monotherapy towards combination therapies has been advised whereby the short-term combination of PMM and MIL was already shown to be a safe and efficacious alternative [24]. However, concerns regarding drug resistance have already been raised for both PMM and MIL monotherapy [10–14, 17, 22, 25]. Given these concerns a better understanding of the possible implications of large scale implementation of PMM-MIL combination therapy seems essential. Therefore, the present study aimed at evaluating the efficacy of PMM-MIL combination therapy on *L*. *infantum* both *in vitro* and in the *in vivo* VL hamster model. Additionally, repeated simultaneous exposure to high doses of PMM and MIL was performed *in vivo* to evaluate the likelihood of PMM- or MIL-resistance development upon combination therapy.

Given the difficult adaptation of clinical isolates maintained *in vitro* for some time, the *in vivo* adapted laboratory strain MHOM/MA/67/ITMAP263 was used for the selection of drug resistance *in vivo*. For the *in vitro* work, the clinical isolate LEM3323 was used as this was the only strain so far for which resistance towards both MIL and PMM could already been demonstrated upon monotherapy *in vitro* [11]. The clear difference in intrinsic susceptibility between both strains is in line with inter-strain variability reported for different parasite species and clinical isolates [26–30]. Although the selection of MIL resistance on amastigotes proved to be very challenging *in vitro*, it could be hypothesized that the enhanced fitness profile associated with PMM resistance could facilitate development of resistance towards other drugs [17]. Our susceptibility analyses did not reveal any direct cross-resistance between PMM and MIL in the individual resistant lines. Despite the fairly rapid selection of PMM resistance upon monotherapy both *in vitro* and *in vivo* [11, 14], no decrease in drug susceptibility could be observed when PMM was administered in combination with MIL either *in vitro* or *in vivo*. This contrasts with the findings of Garcia-Hernandez *et al*., who were able to select resistance to MIL-PMM combinations on *L*. *donovani* promastigotes [31]. This discrepancy is undoubtedly linked to the stage-dependent outcome of the selection procedure for PMM where selection on amastigotes proved to result in parasites with a susceptible promastigote phenotype [13], indicating that the induced mechanisms of resistance are probably different. Contrary to our previous *in vivo* resistance selection experiments during which the parasites were exposed to monotherapy of PMM at 350 mg/kg/day x 5 days or to MIL at 20 mg/kg/day x 5 days [14], the dose regimen in the present study (PMM at 350 mg/kg/day and MIL at 20 mg/kg/day x 2 days) proved to be poorly effective with only 59.3% reduction of parasite burden in the liver, 56.1% in the spleen and 32.5% in the bone marrow (Table 1). Comparable to the field situation where treatment duration was reduced from 28 days MIL at 100 mg/kg/day [32] or 21 days PMM at 11 mg/kg/day [33] to 10 days combination therapy (MIL 100 mg/kg/day + PMM 15 mg/kg/day) [8], we also opted to reduce the treatment duration by about half in the current experimental design. While this resulted in better efficacy in man [8], this approach was clearly inferior to drug monotherapy in the Syrian golden hamster model. On the contrary, combining sub-curative doses of both drugs while maintaining the same treatment duration as in monotherapy resulted in an enhanced efficacy, which is in agreement with previous reports [34]. The relatively moderate *in vivo* activity of PMM at near-toxic doses (350 mg/kg/day) reported here (85.1% reduction in liver, 74.4% in spleen and 84.5% in bone marrow) was already reported previously [14, 35]. Unlike the respective monotherapies, the combination therapy resulted in equally efficient reductions of splenic and hepatic parasite burdens. Although the latter combined dosing schedules showed an excellent efficacy, their high efficacy would severely delay the onset of relapse and complicate repeated relapse/treatment cycles.

The *in vitro* interaction between PMM and MIL on the recent clinical *L*. *infantum* isolate LEM3323 seemed to be indifferent, corroborating observations made for *L*. *donovani* [35]. Although both a MIL- and a PMM resistant phenotype could be generated *in vitro* within 5 drug selection cycles [13, 21], no shift in susceptibility was observed upon *in vitro* drug combination exposure (Table 2). Similar to MIL resistance selection on other strains, a shift in promastigote back-transformation could be observed, indicating that parasites were able to resist elevated drug concentrations [11]. In case of MIL monotherapy, this observation could not be linked to phenotypic parasite changes in drug susceptibility or changes in treatment outcome [36], leaving its clinical relevance still debatable.

Despite several concerns in the past, the results of this study support the view that MIL-PMM combination therapy can be an effective and appealing choice of treatment in view of the fact that the anticipated rapid development of PMM resistance appears to be delayed in combination treatment with MIL.
